# Exploring Job Stress Among Public Health Workforce in Northeastern Malaysia

**DOI:** 10.7759/cureus.49083

**Published:** 2023-11-19

**Authors:** Amer Taufek Abd Wahab, Suhaily Mohd Hairon, Mohd Nazri Shafei, Mohd Ismail Ibrahim, Noriah Mahmud

**Affiliations:** 1 Department of Community Medicine, School of Medicine, Universiti Sains Malaysia, Kota Bharu, MYS; 2 Environmental and Occupational Health Unit, Terengganu State Health Department, Ministry of Health Malaysia, Kuala Terengganu, MYS

**Keywords:** proportion, malaysia, social support, public health workforce, job stress

## Abstract

Introduction

Job stress is recognized as a significant concern across various occupational settings which have profound implications for both individuals and organizations. During the COVID-19 pandemic, job stress among the public health workforce (PHW) has been a significant concern, as they face a significantly increased risk of infection and mortality due to excessive COVID-19 exposure. This study presents a descriptive exploration of key job stress-related factors among PHW in Terengganu, Malaysia.

Methodology

This is a cross-sectional study conducted from May 2022 to April 2023, encompassing all eight District Health Offices (DHO) and government health clinics in Terengganu. Data collection involved a Malay version of the Job Content Questionnaire (M-JCQ), which assesses job characteristics based on Karasek's demand-control-support model and consists of four main domains: decision latitude, psychological job demands, physical job demands, and social support. Data was collected from 1044 participants, and statistical analysis was performed using SPSS version 27 (IBM Corp., Armonk, NY, USA).

Findings

Of the 1044 participants, 18.9% experienced job stress. The highest percentage of job stress was observed among nurses (24.3%), followed by medical assistants (18.3%) and physicians (16.0%). In contrast, the public health assistant (PKA) group had the lowest rate of job stress. Job types based on Karasek's model also showed variations, with doctors having the highest percentage of active jobs (46.4%), while medical assistants had the highest percentage of low job strain (17.9%), and PKAs had the highest percentage of passive job types (44.7%).

Conclusion

This study offers significant insights into the work-related challenges faced by the public health workforce in Terengganu, Malaysia, especially during the COVID-19 pandemic. Job stress is a substantial concern, and understanding its underlying factors is essential for improving the well-being of PHWs. Decision latitude, job demands, and social support play critical roles in shaping job stress among PHWs. Strategies and interventions are needed to mitigate job stress, improve working conditions, and enhance the effectiveness of PHWs in public health initiatives. This study highlights the importance of addressing job stress in this sector, with potential benefits for both the mental and physical health of PHW.

## Introduction

In a broad sense, the public health workforce (PHW) consists of all persons who devote a considerable portion of their time to work that promotes the health of individuals. Specifically, the workforce consists of individuals employed by public health agencies at all levels of government, community-based and non-profit organizations with a health promotion focus [1]. They play a pivotal role in shaping the well-being of communities, addressing health disparities, and safeguarding the health of populations. The nature of their work is multifaceted and demanding, as they engage in activities ranging from disease prevention to health promotion and crisis management. In Malaysia, PHW consists of public health medicine specialists, medical officers (MO), health educators, microbiologists, medical assistants (MA), nurses, health inspectors (IK), epidemiological officers, public health assistance (PKA) and others. Subsequently, this professional category is subdivided between healthcare personnel who are involved in direct patient care (clinical) and those who are not (non-clinical). IK and PKA for example are non-clinical personnel, however, their role as field health investigators allows them to have direct contact with the community.

The concept of job stress is recognized as a significant concern across various occupational settings which have profound implications for both individuals and organizations. Job stress can be defined as the harmful physical and emotional responses that occur when the requirements of the job do not match the capabilities, resources, or needs of the worker [2]. The impact of work stress extends beyond job satisfaction in which it also may affect both general health and job performance because there is a study that showed the job strain was associated with an increased risk of cardiovascular mortality [3]. In the field of public health, where the consequences are significant, it is crucial to comprehend the elements that contribute to work stress and job strain.

During the COVID-19 pandemic, job stress among PHW has been a significant concern, as they face a significantly increased risk of infection and mortality due to excessive COVID-19 exposure [4]. Studies have shown that healthcare workers have experienced a range of psychological outcomes, including depression, anxiety, stress, and psychological distress [5]. Occupational stress, job satisfaction, and intent to leave have also been assessed among PHW dealing with suspected COVID-19 patients, highlighting the impact of the pandemic on their well-being [6]. Social support has been found to play a crucial role in mitigating stress levels among PHW during the pandemic [7]. Coping mechanisms have been explored as strategies utilized by PHW to reduce stress and improve mental health during the pandemic [8].

This study presents a descriptive exploration of key job stress-related factors among PHW in Terengganu, Malaysia. Specifically, we aim to provide an overview of job strain, decision latitude, job demands and social support within this context. By offering a comprehensive snapshot of these aspects, we seek to contribute valuable insights into the working conditions and challenges faced by PHW in Terengganu.

## Materials and methods

This is a cross-sectional study conducted from May 2022 until April 2023 which involved all eight District Health Offices (DHO) and government health clinics in Terengganu. This study used a primary data where the data was collected from the participants by using a Malay version of the Job Content Questionnaire (M-JCQ), which was validated in the study among nurses in Klang Valley, Malaysia in 2015 [7]. This is a well-established self-reported instrument used globally to evaluate job characteristics based on Karasek's demand-control-support model. The M-JCQ consists of four main domains, with a total of 41 items. The domains measured in this tool are decision latitude, psychological job demands, physical job demands and social support. Each of the items has four choices on a Likert scale: strongly disagree, not agree, agree, or strongly agree. The score for the M-JCQ was calculated according to formulas for job content instrument construction based on the guidelines from the Job Content Questionnaire and User’s Guide [8]. In this guideline, the median of the total score was calculated and marked as the cut-off point to distinguish between the low and high categories. Then, the overall distribution of HCWs was classified based on Karasek's job types which were:

1) Active jobs (high psychological demands and high level of job control)

2) Passive jobs (low psychological demands and low level of job control)

3) Low-strain jobs (low psychological demands and high level of job control)

4) High-strain jobs (high psychological demands and low level of job control)

Those who belonged to the high-strain group were defined as having job stress, which meant that they had low job decision latitude in combination with high job demand. The M-JCQ license for the study was approved by the JCQ Centre Global (License no: 18073753480) on 22nd March 2023. The initial sample size required for this research is 916 which was calculated by using a sample size calculator for two independent proportions (P0 was 57% [9], P1 was 66%, alpha was 5% and the power of the study was 80%). However, after considering a 20% dropout rate, the sample size increased to 1145. The PHW from all eight District Health Offices (DHO) in Terengganu were selected using a multistage sampling technique coordinated by the principal investigator. From each DHO, 26 IK and 26 PKA were selected by using computer-generated random sampling, whereas in each district, 30 MO, 30 MA and 30 nurses were selected by simple random sampling from government health clinics (KK) under each DHO. Only PHWs from the primary health sector which are doctors, medical assistants, nurses, health inspectors and public health assistants (PKA) from both government district health offices and government health clinics with at least one year of working experience were included. All foreigners and other occupational groups in primary healthcare such as dieticians, engineers, pharmacists and management staff were excluded from this study. In addition, those PHWs who had psychiatric illnesses and are currently under psychiatry follow-up were also excluded from this study. In this study, PHW was defined as public health workers who worked in a primary healthcare sector under the government. Even though there are many occupational groups involved in this sector, the study only focused on occupational groups that have direct healthcare service to the patients and community as stated in the inclusion criteria. Other operational definition:

1) Psychological Job Demand refers to the mental or cognitive aspects of a job among PHWs that require sustained mental effort in managing tasks at work.

2) Physical demand refers to the physical aspect required by PHWs to perform daily work tasks.

3) Decision latitude refers to the degree of control in each PHW in making decisions about their work.

4) Social support in the workplace involves the perceived availability of help and support to PHWs from colleagues and supervisors.

Each participant provided their informed consent to the researcher before answering any questions. The survey data was then extracted into Excel for analytical purposes and will be exported into SPSS version 27 for further analysis. The analysis used in this study was descriptive analysis and chi-square analysis to explore the job stress and its relationship to occupational groups among PHWs in Terengganu, Malaysia. Ethical approval was granted by Jawatankuasa Etika Penyelidikan Manusia (JEPeM) of Universiti Sains Malaysia and Malaysia Research and Ethic Committee’s (MREC) [NMRR ID-22-01743-LTR (IIR)], Ministry of Health Malaysia prior to the data collection.

## Results

A total of 1044 participants were involved in this study where the highest number of respondents among PHWs were medical assistants (23.6%), followed by medical officers that were 22.7%. The lowest number of respondents were from the public health assistant (PKA) group which was 13.5%. The mean (SD) age was 37.24 (7.64) years. Out of 1044 participants, the female and male PHWs were 528 (50.6%) and 516 (49.4%) respectively. The mean (SD) for working experience was 12.65 (7.48) years. Other than that, most of the participants were from health clinics which is 648 (62.1%) of the total PHWs. Table 1 summarizes the sociodemographic characteristics of PHWs in Terengganu.

**Table 1:** Sociodemographic characteristics of the Public Health Workforce (PHW) in Terengganu (n = 1044) SPM = Sijil Pelajaran Malaysia (secondary education) SD = Standard deviation

Variables	Frequency (%)	Mean (SD)
Age		37.24 (7.64)
Gender		
Male	516 (49.4)	
Female	528 (50.6)	
Marital status		
Single	126 (12.1)	
Married	918 (87.9)	
Education		
SPM	213 (20.4)	
Diploma	531 (50.9)	
Degree	285 (27.3)	
Master/PhD	15 (1.4)	
Occupational Group		
Medical officer	237 (22.7)	
Medical Assistant	246 (23.6)	
Nurses	214 (20.5)	
Health Inspector	206 (19.7)	
Public Health Assistant	141 (13.5)	
Working Experience		12.65 (7.48)
Working in Shift		
Yes	15 (1.4)	
No	1029 (98.6)	
Workplace		
Health Office	396 (37.9)	
Health Clinics	648 (62.1)	

Table 2 provides a summary of the distribution of scores throughout JCQ domains. In addition to the formula for job content instrument construction, the decision latitude score was calculated from a total of nine questions that produced a broad range of scores from 11 to 36 with a mean (SD) score of 27.59 (3.95). The range of scores for psychological job demand is 13 to 32, with a mean (SD) of 22.91 (3.11). In contrast, the minimal score for physical job demand is 5 and the maximum score is 20, with a mean (SD) score of 11.4 (3.00). The range of possible social support domain scores is 11 to 32, with a mean (SD) score of 25.47 (3.77). The scores ranged from 4 to 16 for supervisor support and 5 to 16 for coworker support, with a mean (SD) for each domain of 12.09 (2.46), and 13.39 (2.18), respectively.

**Table 2:** Distribution of the scores according to the job content questionnaires domain among Public Health Workforce (PHW) in Terengganu. SD = Standard deviation

Variable	Minimum Score	Maximum Score	Mean (SD)
Decision Latitude	11	36	27.59 (3.95)
Skill Discretion	8	24	19.83 (2.93)
Decision Authority	3	12	7.76 (1.65)
Psychological job demand	13	32	22.91 (3.11)
Physical Job Demand	5	20	11.40 (3.00)
Social Support	11	32	25.47 (3.77)
Supervisor Support	4	16	12.09 (2.46)
Co-worker Support	5	16	13.39 (2.18)

In general, out of 1044 PHWs involved in this study, 197 (18.9%) of them had job stress. Table 3 shows the distribution of the Karasek job types according to their occupational group. Statistically, this research revealed a significant association between Karasek's job type and occupational group. In addition, it was shown that the nurse's group had the highest percentage of occupational stress at 24.3%, followed by medical assistants and physicians at 18.3% and 16.0%, respectively. On the contrary, the public health assistant (PKA) group had the lowest rate of occupational stress. In another perspective of Karasek job types, doctors were found to have the highest percentage of active jobs with 46.4%, whereas medical assistants were found to have the highest percentage of low job strain with 17.9%, and PKA had the highest percentage of passive jobs type with 44.7%.

**Table 3:** The distribution of the Karasek's job types based on their occupational group among Public Health Workforce (PHW) in Terengganu (n = 1044) IK = Health Inspector; PKA = Public Health Assistant; MA = Medical Assistant * Pearson chi-square statistics

Occupational group (%)	Karasek Job Types				X^2^ (df)	P-value
	Active Jobs	Job Stress	Low Job Strain	Passive Jobs		
PKA	36 (25.5%)	24 (17.0%)	18 (12.8%)	63 (44.7%)	45.275 (12)	<0.001
IK	66 (32.0%)	38 (18.4%)	21 (10.2%)	81 (39.3%)		
Nurse	77 (36.0%)	52 (24.3%)	28 (13.1%)	57 (26.6%)		
MA	96 (39.0%)	45 (18.3%)	44 (17.9%)	61 (24.8%)		
Doctor	110 (46.4%)	38 (16.0%)	35 (14.8%)	54 (22.8%)		

## Discussion

The present study aimed to provide a comprehensive exploration of key job-related factors among the public health workforce (PHW) in Terengganu, Malaysia. The findings shed light on the levels of job stress, decision latitude, job demands, social support, and job strain within this context. In this study, we found that the prevalence of job stress among the PHWs in Terengganu was slightly lower than in another study among healthcare workers (HCWs) by Ab Aziz et al. [9] in Kelantan, Malaysia where the incidence of job stress was 28.5%. This could be attributed to the fact that PHWs in Terengganu have substantial social support from their employers and coworkers. The results of this study indicate that job stress is a significant concern among PHWs in Terengganu. This is consistent with previous research highlighting the impact of the COVID-19 pandemic on the mental health and well-being of healthcare workers [5].

Decision latitude, which refers to the level of control and autonomy that individuals have in their work, is an important factor to consider in relation to job stress. Based on Karasek's demand-control-support model, low decision latitude will lead to job stress when the psychological job demand is high [10]. The findings of this study suggest that PHWs in Terengganu have a lower level of decision latitude, with a mean score of 27.59 out of a possible range of 11 to 36 as compared to another study in the east coast of Malaysia which has a higher mean score of decision latitude [9]. This indicates that PHWs have some level of control over their work, but there may be room for improvement in terms of providing them with more decision-making authority and autonomy to avoid getting job stress.

Job demands, both physical and psychological, are also important factors to consider in relation to job stress among PHWs. The findings of this study indicate that PHWs in Terengganu experience moderate levels of psychological job demands, with a mean score of 22.91 out of a possible range of 13 to 32. This suggests that PHWs face significant high job demand in their work which may be caused by the lack of health staff at work which in turn will cause the health staff to experience job stress and further impact the health service [11]. It is important for the Ministry of Health of Malaysia (MOH) as their employer to provide adequate support and resources to help PHWs cope with these demands and reduce their stress levels.

Social support is another crucial factor in mitigating job stress among PHWs. The findings of this study suggest that PHWs in Terengganu have a moderate level of social support, with a mean score of 25.47 out of a possible range of 11 to 32. This indicates that PHWs perceive some level of support from their supervisors and coworkers, but there may be room for improvement in terms of enhancing social support networks within the workplace. Previous research has shown that social support plays a crucial role in buffering the negative effects of job stress and promoting well-being among healthcare workers [7]. A study by AbuAlRub [12] and Ab Aziz et al. [9] on healthcare professionals has shown that receiving social support from either a supervisor or coworkers may effectively alleviate job-related stress. Therefore, efforts should be made to strengthen social support systems for PHWs in Terengganu.

The concept of job strain, which refers to the combination of high job demands and low decision latitude, is an important consideration in relation to the well-being of public health workers (PHWs) [13]. Nurses in Terengganu have the highest percentage of job strain, followed by medical assistants and doctors. This indicates that nurses may be particularly vulnerable to experiencing high levels of job stress due to the combination of high job demands and limited decision latitude [14]. Thus, addressing this issue and providing support and resources to help nurses cope with their demanding work environment is crucial. A study by Khamisa et al. [15] emphasizes the importance of developing strategies and intervention programs to improve nurse and patient-related outcomes. The implications of job stress extend beyond psychological well-being. According to research conducted by Kivimäki et al. [16], job-related stress is associated with an increased risk of cardiovascular death. This underscores the significance of addressing job distress not only for the mental health of PHWs but also for their general physical health in the long term [3].

Limitations

Although the JCQ has commendable qualities, it is important to acknowledge its underlying limitations. The JCQ depends on self-reported data provided by workers, including a subjective component. Employees' opinions of their work environment might differ, and their replies may be impacted by their own interpretations and experiences. Hence, it is essential to acknowledge that JCQ findings are indicative of the subjective perspectives of workers. In addition, the self-report data provided by workers may be subject to response bias. Employees may exhibit reluctance to articulate adverse emotions or apprehensions about their work milieu, due to apprehension of possible consequences. Alternatively, individuals may also be tempted to react in a way that corresponds to their perception of their employer's expectations, resulting in response bias and erroneous outcomes.

To overcome the limitations of the Job Content Questionnaire (JCQ) in future research, it is recommended to integrate it with supplementary evaluation tools and methodologies. Interviews, observations, and objective measurements of workload or job performance may offer additional perspectives and provide a more comprehensive understanding of the work environment. It is advisable to modify the JCQ questionnaire to better suit the particular setting, cultural context, or professional environment of the personnel under evaluation. Tailoring the questions and scaling can significantly improve the relevance and precision of the gathered data. In addition, it is imperative to guarantee the confidentiality of workers' responses to effectively counteract response bias. Furthermore, it is of paramount importance to explicitly assure employees that their input will not be used to their detriment, thereby fostering an atmosphere of trust and transparency.

## Conclusions

In conclusion, the notion of job strain is defined by the combination of high job demands and low decision latitude, while also being influenced by the presence of social support. Thus, it is a significant consideration for the well-being of PHWs. Therefore, it is a crucial factor to consider for the well-being of PHWs. Nurses, specifically, may be susceptible to elevated levels of job-related stress as a result of the combination of these factors. It is vital to address job strain and provide support and resources to help PHWs, particularly nurses, manage their demanding work environment. By implementing strategies and intervention programmes, it is possible to mitigate the negative effects of job distress, resulting in enhanced well-being and performance among PHWs.

In addition, this study provides valuable insights into the working conditions and challenges faced by PHWs in Terengganu. The findings highlight the importance of addressing job stress, enhancing decision latitude, managing job demands, strengthening social support networks, and addressing job strain among PHWs. These findings have implications for the well-being and effectiveness of PHWs in their roles as key players in public health initiatives. Future research should focus on developing and implementing interventions to address these issues and promote the well-being of PHWs in Terengganu.
